# Reduced plasma concentrations of vitamin B6 and increased plasma concentrations of the neurotoxin 3-hydroxykynurenine are associated with nodding syndrome: a case control study in Gulu and Amuru districts, Northern Uganda

**DOI:** 10.11604/pamj.2016.24.123.8409

**Published:** 2016-06-08

**Authors:** James Henry Obol, Denis Anywar Arony, Ronald Wanyama, Kenneth Luryama Moi, Bongomin Bodo, Patrick Olwedo Odong, Michael Odida

**Affiliations:** 1 Department of Public Health, Faculty of Medicine, Gulu University, P.O Box 166, Gulu; 2 Department of Medical Biochemistry, Faculty of Medicine, Gulu University, P.O Box 166, Gulu; 3 Department of Microbiology and Immunology, Faculty of Medicine, Gulu University, P.O Box 166, Gulu; 4 Department of Paediatrics and Child Health, Faculty of Medicine, Gulu University, P.O Box 166, Gulu; 5 District Health Office, Amuru District Local Government, P.O Box 1074 Gulu; 6 Department of Pathology, Makerere University College of Health Sciences, P.O Box 7072 Kampala

**Keywords:** Nodding syndrome, case-control study, vitamin B6, 3-hydroxykynurenine, Northern Uganda

## Abstract

**Introduction:**

Nodding syndrome was first reported in Uganda in 2003 among internally displaced populations. Risk factors for the syndrome remain unknown. We therefore explored vitamin B6 deficiency and resulting high 3-hydroxykynurenine (3-HK) levels as risk factor for nodding syndrome in Northern Uganda.

**Methods:**

Case-control study conducted in Gulu and Amuru districts. Cases were children/young adults with nodding syndrome. Healthy children/young adults were recruited as controls from same community as cases. Data on socio-demographic and other risk factors was collected using questionnaires. Whole blood was collected in EDTA tubes for assay of 3-HK and vitamin B6 using sandwich ELISA. Conditional logistic regression model was used to assess associations.

**Results:**

66 cases and 73 controls were studied. Factors associated with nodding syndrome were being positive for 3-HK (AOR=4.50, p=0.013), vitamin B6 concentration below mean (AOR=7.22, P=0.001), child being taken care of by mother only (AOR=5.43, p=0.011), child being taken care of by guardian (AOR=5.90, p=0.019) and child consuming relief food at weaning (AOR=4.05, p=0.021).

**Conclusion:**

Having low vitamin B6 concentration which leads to a build up of 3-hydroxykynurenine concentration in cases as a main risk factor. Therefore, cases should be treated with vitamin B6 and community members should be sensitise to ensure adequate dietary intake of vitamin B6 so that the risk of nodding syndrome among children is averted. We encourage future prospective intervention study to be conducted to assess the effect of low vitamin B6 on the development of nodding syndrome via raised 3-HK concentration.

## Introduction

Nodding syndrome was first described in Tanzania in the 1960s and similar conditions were reported in the 1980s in the southern part of Sudan (presently the Republic of South Sudan) [1, 2]. It is currently unknown what causes nodding syndrome [3], which can be fatal or cause mental and physical disability in young children between the ages of 5 and 15 [4, 5]. In Uganda, the syndrome is currently restricted to the northern region where about 5,000 cases have been detected and an estimated 300 children have succumbed to it [4]. Nodding syndrome was first reported in Uganda around 1997 among the population of internally displaced people's (IDP) camps [6]. The onset of symptoms in affected children is marked by the development of nodding head movements, which are reported to be provoked either by the sight of food or exposure to cold weather [4, 7]. The nodding head movements have been documented to correspond with the onset of subclinical brain seizure activity on electroencephalography (EEG) [7]. Seizure activity is brief and halts when the child stops eating or feels warm again [4]. The syndrome is progressive [3, 4] and is associated with cognitive impairment, stunting, lip changes and other physical deformities [7], mental retardation, behavioural disabilities and malnutrition [4, 6]. Magnetic resonance imaging (MRI) scans have shown atrophy of cortical, cerebellar [3, 7], hippocampal [3] and glial cells of cases [8]. There is to date no report of recovery from nodding syndrome; many cases have died from the syndrome [9]. Several etiological factors have been proposed, including infec¬tious, nutritional, environmental, and psychogenic causes [3, 10]. Specific suspected causal exposures which were evaluated in previous studies include Onchocerca volvulus, bombs dropped during wartime, nutritional deficiencies, measles infection, consumption of monkey meat, and consumption of possibly contaminated relief seeds and relief food [11]. However, the exact cause and the pathophysiology of nodding syndrome remain unknown [1, 4]. Low level of vitamin B6 was reported among cases and control in one study [10] and testing has failed to demonstrate associations between nodding syndrome and vitamin B6 [10, 12]. We hypothesised that vitamin B6 deficiency resulting into increased production of neurotoxic 3-HK could predispose to nodding syndrome. This hypothesis was based on a previous study demonstrating that vitamin B6 deficiency leads to abnormal tryptophan metabolism, with the metabolite excreted in greatest quantity being 3-HK [13]. 3-HK, an endogenous tryptophan metabolite, is known to have toxic effects in the brain cells [14]. This study was therefore set in the context of this prevailing knowledge gap to explore vitamin B6 deficiency and resulting high 3-HK levels as a risk factor for nodding syndrome in the Acholi sub-region of Northern Uganda.

## Methods

**Study area:** The study was conducted in Gulu and Amuru districts in Northern Uganda. In Gulu district the study was conducted in Paicho sub-county; Omel Parish while in Amuru district the study was conducted in Atiak sub-county; Okidi and Pachilo parishes. The study areas were chosen purposively because nodding syndrome cases are localised in those areas. The two districts are post conflict areas which have experienced over two decades of armed insurgency.

**Sample size estimation, research participant recruitment, sampling procedure and data collection:** We used formula for calculating sample size for a proportion in two groups [15] with alpha level of 0.05, estimated difference in the two proportions was 0.2 and power set at 90%. This gave a minimum sample size of 60 in each group. Our inclusion criterion was being a confirmed case of nodding syndrome while the exclusion criteria were unconfirmed case status or epilepsy. The first International Scientific Meeting on Nodding Syndrome held in Kampala, Uganda in 2012 adopted the definition of a confirmed case as “a probable case plus a documented nodding episode that was observed by a trained healthcare worker”. A probable case was defined as a suspected case with both of the following major criteria: age at onset of nodding between 3 and 18 years old; frequency of nodding 5 to 20 per minute; plus at least one of the following minor criteria: other neurological abnormalities (cognitive decline, school dropout due to cognitive/behavioral problems, other seizures or neurological abnormalities);clustering in space or time with similar cases; triggered by food and/or cold weather; stunting or wasting, delayed sexual or physical development, psychiatric symptoms [16]. All case-control subjects were formerly internally displaced people living in IDP camps who had returned to their ancestral home. Village health team (VHT) members in the studied communities were contacted to mobilise parents to assemble with their children (both cases and controls) in one central location prior to our visit to the community. Cases were those previously confirmed after examination by a trained healthcare worker and were enrolled on treatment/nutritional supplementation. Both cases and controls were recruited in the study from their communities. Controls were healthy children/young adults of similar age group as cases. Participants were recruited between 12^th^ November and 11^th^ December 2013. A questionnaire was used to collect data on socio-demographic characteristics and other possible risk factors. Venous blood was collected in EDTA tubes and transported to the laboratory in an icebox. Blood was centrifuged immediately on arrival in the laboratory and aliquots were stored at -200C until analysis.

**Laboratory procedures for 3-hydroxykynurenine and vitamin B6 assay:** We used enzyme-linked immunosorbent assay (ELISA) methods that employ quantitative sandwich enzyme immunoassay techniques. The 3-HK assay has a detection range of 78 pmol/ml- 5000 pmol/ml [17] while the vitamin B6 assay has a detection range of 5pmol/l- 1500pmol/l [18]. Assays were performed using standard procedures as prescribed by the manufacturer. We performed the following internal quality control among which include priming ELISA washer prior to washing test plates; rinsing washer using distilled water; calibrating ELISA reader using blank solution before reading; Duplicate running of blank solution, negative controls and positive controls. The averages of positive controls were used to construct the standard curve. Reading of blank solution well was compared with readings of negative controls to see if the test was performed accurately and were taken as zero.

**Data management and analysis:** Curve Expert 1.4 [19] was used to construct a standard curve using optical density (OD) values for the standards. This standard curve was then used to calculate the concentration of 3-HK and vitamin B6 in samples. The questionnaire data on each case and control plus the 3-HK and Vitamin B6 concentrations were then entered into EpiData 3.1 and exported to STATA 10 for analysis. Continuous variables were summarised using means and categorical data were reported as proportions. Spearman's rank correlation coefficient (r_s_) was used to assess correlation between vitamin B6, 3-HK concentration in the plasma and Source of food consumed at weaning. Conditional logistic regression model was used to assess possible associations between nodding syndrome and socio-demographic characteristics or laboratory findings. Crude odds ratio and age-adjusted odds ratios plus 95% confidence intervals (CI) for the independent variables were calculated. The final model was age adjusted because cases were significantly older than controls in our sample. Any independent variable with p-value > 0.05 was taken as statistically significant and thus associated with nodding syndrome.

**Ethical considerations:** The study was approved by the Institutional Review Board, Gulu University and ethical clearance was granted by the Uganda National Council for Science and Technology. The background, risks, benefits, procedures to be performed and the right to decline participation in the study were explained before written informed consent was obtained from cases that were able to provide consent and from all controls. Each participant's parent/guardian provided written informed consent after explanation of the background to the study, procedure to be performed on their child, risks and benefits before any information was obtained or procedure was performed on their child. All information obtained from study participants or their parents/guardians for this study is being treated with strict confidentiality.

## Results

**Figure 1:**
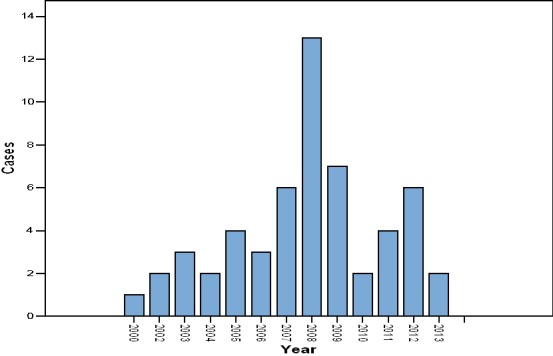
Reported year of nodding syndrome onset for 55 out of 66 cases by parents/guardians

**Figure 2:**
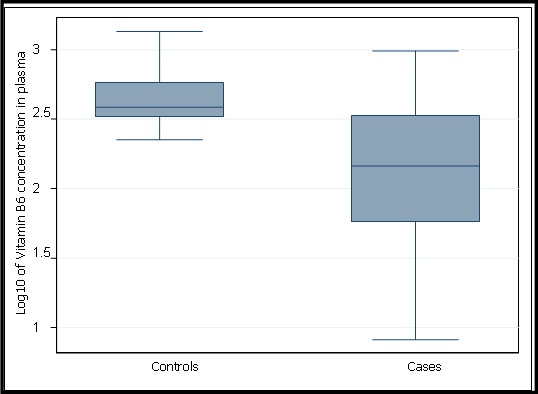
Box and Whisker plot for lo10 of Vitamin B6 concentration in pmol/l in plasma

**Figure 3:**
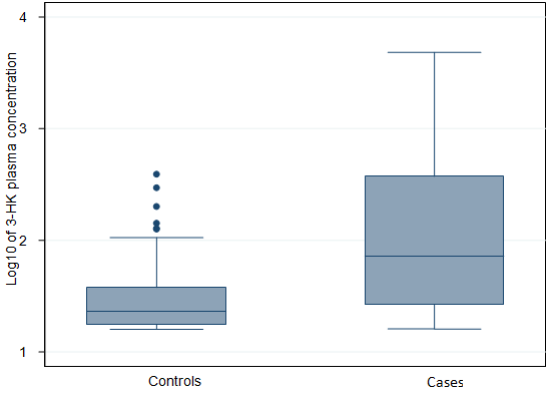
Box and whisker plot for log10 of 3-HK concentration in pmol/ml in plasma

We recruited 66 cases and 73 controls in the study. Of the 66 cases, 55 parents/guardians were able to recall the year of nodding syndrome onset (Figure 1). 46 (70%) cases and 34 (47%) controls were male. The ages of the research participants ranged from 2 to 21 years. Cases were older than controls; the mean age was 12.5 years for cases and 8.9 years for controls. 23% of cases were born at health facilities compared with 83% of controls. 41% of cases and 76% of controls were cared for by both parents. Table 1 summarises the demographic characteristics of the study participants. Vitamin B6 concentration was negatively correlated with 3-HK concentration in the plasma of study participants (r_s_ = -0.366, p <0.001) and Source of food consumed at weaning (r_s_ = -0.325, p<0.001). The mean concentration of vitamin B6 was 202.8pmol/l (95% CI 159.3-246.2) in cases and 512.8pmol/l (95% CI 446.9-578.7) in controls. The mean concentration of 3-hydroxykynurenine was 466.4pmol/ml (95% CI 228.9-703.9) in cases and 45.3pmol/ml (95% CI 31.0- 59.6) in controls. Figure 2 shows Log10 of 3-HK concentration in pmol/ml in plasma for cases and controls. Figure 3 shows Log10 of Vitamin B6 concentration in pmol/l in plasma of cases and controls. Factors associated with nodding syndrome were:- being taken care of by mother only (AOR=5.43, 95% CI 1.48 - 19.87, p=0.011), being taken care of by guardian (AOR=5.90, 95% CI 1.34 - 25.93, p=0.019), consumption of relief food at weaning (AOR=4.05, 95% CI 1.23 - 13.28, p=0.021), Having vitamin B6 concentration below the mean in plasma (AOR = 7.22, 95% CI 2.24 - 23.26, P=0.001) and Having detectable 3-HK in plasma (AOR=4.50, 95% CI 1.37 - 14.77, P=0.013). Table 2 summarises the results for crude odds ratio and age-adjusted odds ratio for socio-demographic characteristics associated with nodding syndrome.

**Table 1:** Socio-demographic characteristics of the study participants

-	Status of study participants	
Characteristics	Cases = 66 (47%)	Controls= 73 (53%)
Gender		
Female	20 (30)	39 (53)
Male	46 (70)	34 (47)
Age (mean)	12.5	8.94
Age groups		
2 - 10 Years	16 (25)	50 (69)
11 - 21 Years	48 (75)	22 (31)
Care taker of the child		
Father & Mother	26 (41)	55 (76)
Father	2 (3)	1 (1)
Mother	21 (33)	10 (14)
Guardian	14 (22)	6 (8)
Age group of lead care taker		
21 - 39 Years	28 (44)	47 (65)
40 - 80 Years	35 (56)	25 (35)
Child place of birth		
Health unit	14 (23)	60 (83)
At home	47 (77)	12 (17)

**Table 2:** Crude Odds Ratio (COR) and age-adjusted Odds Ratio (AOR) for socio-demographic characteristics associated with Nodding syndrome

	Status of study Participants							
Variables	Cases = 66 (47%)	Controls = 73 (53%)	COR	95% CI	P-value	AOR	95% CI	P-value
**3-Hydroxykynurenine**								
Negative (below detection limit)	34 (52)	61 (84)	1			1		
Positive (above detection limit)	32 (48)	12 (16)	4.78	2.18 - 10.49	**≤0.001**	4.50	1.37 - 14.77	**0.013**
**Vitamin B6 level in plasma (pmol/l)**								
Above mean (365.603 - 1350.36)	8 (12)	42 (58)	1			1		
Below mean (8.15 - 365.602)	58 (88)	31(42)	9.82	4.10 - 23.51	**≤0.001**	7.22	2.24 - 23.26	**0.001**
**Gender**								
Female	20 (30)	39 (53)	1			1		
Male	46 (70)	34 (47)	2.63	1.31 - 5.30	**0.006**	2.23	0.78 - 6.34	0.158
**Care taker of the child**								
Father & Mother	26 (41)	55 (76)	1			1		
Father	2 (3)	1 (1)	4.23	0.37 - 48.80	0.248	0.90	0.05 - 15.44	0.943
Mother	21 (33)	10 (14)	4.44	1.83 - 10.77	**0.001**	5.43	1.48 - 19.87	**0.011**
Guardian	14 (22)	6 (8)	4.94	1.70 - 14.30	**0.003**	5.90	1.34 - 25.93	**0.019**
**Child exclusively breast fed**								
No	5 (8)	2 (3)	1					
Yes	55 (92)	69 (97)	0.32	0.06 - 1.71	0.182	0.18	0.02 - 1.65	0.131
**Source of food consumed at weaning**								
Home grown food	20 (33)	49 (73)	1			1		
Relief food	40 (67)	18 (27)	5.44	2.54 - 11.66	**≤0.001**	4.05	1.23 - 13.28	**0.021**
**Mother was feeding well when breastfeeding the child**								
No	22 (36)	10 (14)	1					
Yes	39 (64)	62 (86)	0.29	0.12 - 0.67	**0.004**	0.90	0.27 - 3.00	0.867

## Discussion

Data from our study indicates that cases were much older than controls and some had lived with the syndrome for over ten years. This is consistent with other studies done on similar groups [10, 11]. Nodding syndrome is neurological disorder that becomes more severe with time as reported by other investigators [20, 21]. This explains why cases were much older than controls because cases had lived with the syndrome for many years. Majority of cases were born at home compared with majority of controls who were born at health facilities. At the peak of the insurgency in northern Uganda, majority of the population were displaced living in internally displaced persons’ camps where most health facilities were closed due to insecurity [22]. Majority of controls were born at health facilities because when government health facilities were inaccessible, relief agencies had to provide make-shift health facilities within the IDP camps to serve the IDP population [23]. We found the number of children with nodding syndrome onset to be higher in 2008 than any other year. This corresponds with the time government was trying to disband the IDP camps in northern Uganda [24]. In our study, children who were being taken care of by their mother only were about 5 times more likely to have nodding syndrome than children cared for by both parents. Children who were being taken care of by a guardian were about 6 times more likely to have nodding syndrome compared with children cared for by both parents. Children whose caretakers were their mothers or guardians only may not have an adequate diet, as it may be difficult for single mothers or guardians (in most cases grandmothers without any source of livelihood) to supplement relief food.

The mean vitamin B6 concentration was 2.5 times higher in controls than in cases while the mean plasma concentration of 3-HK was 10 times higher in cases than in controls. The odds of a case having detectable plasma 3-HK was increased by a factor of 4.50 compared to controls, after adjusting for other variables in the model (p=0.013). Also, 88% of cases and 42% of controls had vitamin B6 concentrations below the mean. The adjusted odds of being a case for those having vitamin B6 concentrations below the mean was 7.22 compared with those above the mean (P=0.001). Other studies have reported vitamin B6 deficiency among nodding syndrome cases [10, 12]. A study showed that some substances may lower vitamin B6 levels, including various drugs (cycloserine, hydralazine, isonazid, penicillamine), smoking, alcoholism and consumption of the mushrooms Gyromitra esculenta and Agaricus bisporis [25]. None of the cases were on any of the above drugs or indicated mushrooms as one of their sources of food at the time of study. Smoking and drinking alcohol are not practiced among children and young adult as it is against the cultural norms among the Acholi community of northern Uganda where this study was conducted. However, further analysis of data showed an inverse correlation between vitamin B6 and source of food consumed at weaning. Also, children whose parents indicated that they were fed on relief food at weaning were more likely to experience nodding syndrome than children fed on home grown food at weaning. This finding is in contrast to a result of a study done on nodding syndrome patients in Kitgum district of northern Uganda [10]. Relief foods distributed were mainly maize and bean and these foods are deficient of B6 in them [26]. The first case of nodding syndrome was reported in 2003 among populations living in internally displaced persons’ camps [6] and when most people were relying on relief food. Our study shows an inverse correlation between vitamin B6 and 3-HK and this is consistent with other study [27]. Cases were more likely to have decreased level of vitamin B6 concentration than controls and elevated level of the neurotoxic 3-HK in their plasma than controls. This is consistent with other studies which have shown that vitamin B6 deficiency leads to a build up of 3-HK concentration in human body [13, 28, 29].

Therefore it is important to note whether plasma levels of 3-HK can reflect brain levels of 3-HK. Studies in animal have demonstrated that peripheral 3-HK is actively transported from the circulation through the blood brain barrier [30]. Increased plasma 3-HK levels therefore can lead to an increased brain levels of 3-HK. In other pathological disorders like Parkinson's disease, Alzheimer's disease (AD) and acquired immunodeficiency syndrome dementia [31–33], the concentration of 3-HK have been found to be greatly increased. These diseases are known to be associated with neuronal cell death thereby causing dysfunction in neuronal circuitry [14]. The neurotoxicity of 3-HK on brain cells could explain why other studies using MRI scan had reported brain atrophy among nodding syndrome patients [3, 7, 8]. Our study had the following limitations: Recall bias since most of the parents/guardians were asked about their past events. Parent/guardians of cases may recall better past event for their children than parents/guardians of controls because of the problem they are currently faced with. We also could not compare the level of Vitamin B6 and 3-HK both in cases and control at the point when cases started experiencing nodding syndrome due to the case-control design of the study. Cases were older than controls due to the fact that we match cases to controls based on the age range for nodding syndrome patients as had been reported by other studies to be between 5-15 years [10, 11, 20, 21]. Further more, we did not match cases and control for sex since previous study had shown that excretion of 3-HK is not affected by gender [34]. Also, 52% of the cases did not have raised 3-HK in their plasma and this could be attributed to nutritional supplement including multivitamins which were being given to nodding syndrome cases. Management of malnutrition is one of the key activities in the guidelines for managing nodding syndrome in Uganda as proposed by Ministry of Health [21]. A study has revealed that the concentration of 3-HK was found to dramatically decreased following supplementation with pyridoxine [13]. Other study has shown that vitamin B6 is required as a coenzyme in the catabolism of 3-HK in human body [35]. Thus nutritional supplementation with multivitamins would have provided vitamin B6 which is required to catabolise 3-HK in the cases. This would then lead to depletion of 3-HK in the plasma of cases thereby testing negative for raised 3-HK in plasma. Our findings need to be replicated and expanded in future studies. Despite these limitations, this investigation provides new information in addition to those already published on nodding syndrome.

## Conclusion

We identified that having low vitamin B6 concentration which leads to a build up of 3-HK concentration in cases as a main risk factor. Therefore, cases should be treated with vitamin B6 and community members be sensitised on adequate dietary intake of food rich in vitamin B6 so that the risk of nodding syndrome among children is averted. We encourage future prospective intervention study to be conducted to assess the effect of low vitamin B6 on the development of nodding syndrome via raised 3-HK concentration.

### What is known about this topic

- Nodding syndrome is association with Onchocerca volvulus detected by skin snip or serologic analysis;
- Magnetic resonance imaging (MRI) scans have shown atrophy of cortical, cerebellar, hippocampal and glial cells of cases;
- Head nodding is triggered by sight of food or exposure to cold weather.

### What this study adds

- Cases were having reduced vitamin B6 concentration in their plasma than controls and this is significantly associated with nodding syndrome;
- Cases were having raised concentration of 3-hydroxykynurenine in their plasma than controls and this is significantly associated with nodding syndrome;
- Feeding children on relief food at weaning is significantly associated with nodding syndrome in our study.
